# Effects of High Dose of Atorvastatin for Preventing Periprocedural Ischemic Brain Damage in Patients Undergoing Carotid Artery Stenting (PICAS) in China: A Randomized Controlled Clinical Trial

**DOI:** 10.3389/fneur.2020.00937

**Published:** 2020-08-25

**Authors:** Haipeng Wang, Junjie Wang, Jun Lu, Daming Wang

**Affiliations:** ^1^Department of Neurosurgery, National Center of Gerontology, Beijing Hospital, Beijing, China; ^2^Graduate School of Peking Union Medical College, Beijing, China

**Keywords:** clinical trials, high-dose statin therapy, carotid artery stenting, periprocedural ischemic complication, Chinese ethnicity

## Abstract

**Background:** Research conducted in Western countries has suggested that high-dose statin therapy can lead to the regression of carotid atherosclerotic plaques and can reduce periprocedural ischemic complication rates in individuals undergoing carotid artery stenting (CAS). However, whether this same therapeutic approach is of value in patients of Chinese ethnicity is not as well-established.

**Methods:** This is a single-center, prospective, parallel-controlled, intervention-based efficacy study that will enroll a total of 130 Chinese patients with cervical carotid stenosis who are scheduled to undergo CAS. These patients will be randomly divided into a routine treatment group and a high-dose atorvastatin group. Individuals in the routine treatment group will be administered standard of care 20 mg/day atorvastatin treatment. Individuals in the high-dose atorvastatin group will be administered 80 mg/day atorvastatin for 3 days prior to and following CAS. The primary outcome of this study will be the cumulative incidence of new cerebral ischemic lesions on diffusion-weighted magnetic resonance imaging (DW-MRI) within 5 days following CAS, and of transient ischemic attacks (TIAs) or ischemic stroke within 30 days after CAS.

**Discussion:** This study is the first to assess whether high-dose atorvastatin treatment is capable of reducing the incidence of perioperative cerebral ischemic injury in patients of Chinese ethnicity undergoing CAS. These results will offer evidence regarding which statin treatment regimens are more appropriate when treating Chinese patients undergoing CAS in an effort to minimize their risk of any perioperative cerebral ischemic injury.

**Trial Registration:** ClinicalTrials.gov NCT03079115; registered March 14, 2017.

## Introduction

### Perioperative Ischemic Stroke of CAS

Carotid artery stenting (CAS) is frequently employed to treat patients suffering from stenosis of the carotid artery, but this procedure is associated with a relatively pronounced perioperative risk of ischemic complications, of which up to 90% occur within 7 days following treatment (1, 2). One recent meta-analysis determined that ~40.3% of CAS patients suffer from the development of new cerebral ischemic lesions that are visible upon diffusion-weighted magnetic resonance imaging (DW-MRI) following surgery (3). These patients that exhibit new ischemic foci are in turn more likely to suffer from a subsequent stroke or transient ischemic attacks (TIA) during follow-up (4), and they are more likely to exhibit cognitive decline (5).

### High-Dose Statin Therapy as a Means of Achieving Neuroprotection in CAS Patients

Current guidelines for patients undergoing CAS indicate that patients should receive statins during the perioperative period, but the dosage or duration of such statin administration is not specified (6). The high-dose administration of atorvastatin (80 mg/day) has been found to mediate a number of potentially neuroprotective effects, including enhanced protection of vascular endothelium, blood vessel dilation, and reduced rates of thrombosis. In a study of carotid stenosis patients from Western nations, such high-dose atorvastatin administration was shown to result in a lower risk of the development of new ischemic lesions following CAS surgery, and to be protective for cerebrovascular and brain tissues (7).

As there are potentially significant differences between populations of different genetic backgrounds with respect to their responses to statin therapy, whether high-dose atorvastatin administration is associated with the same benefits in patients of Chinese ethnicity undergoing CAS is not as well-understood. As such, the present article outlines the design of a PICAS study aimed at evaluating the safety and efficacy of high-dose atorvastatin administration during the perioperative period in patients of Chinese ethnicity undergoing CAS. The primary aim of this study is to assess the ability of such high-dose atorvastatin treatment to reduce the incidence of ischemic brain injury in CAS patients of Chinese ethnicity. The secondary aim of this study is to determine whether there are any differences in the safety profiles of normal- and high-dose atorvastatin regimens.

## Methods and Analysis

### Study Design

**Figure 1:**
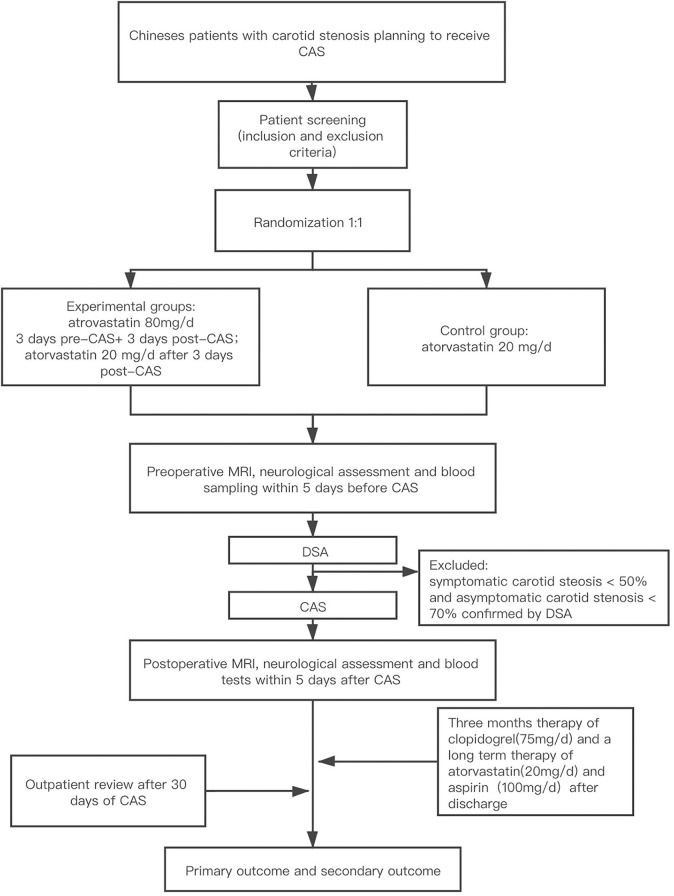
Study design. CAS, carotid artery stenting; MRI, magnetic resonance imaging; DW-MRI, diffusion-weighted magnetic resonance imaging.

This is a single-center, single-blind, prospective, parallel-controlled, intervention-based efficacy study that will enroll 130 consecutive patients suffering from carotid stenosis that are scheduled to undergo carotid artery stenting in Beijing hospital. The study process is summarized in Figure 1.

### Participants

This study will only enroll patients of Chinese ethnicity between the ages of 40 and 90 years that are able to undergo a minimum of 2 weeks of treatment with statin therapy (regardless of type and dose of statin therapy). These patients must exhibit symptoms associated with stenosis of the carotid artery, with symptomatic patients exhibiting ≥50% carotid artery stenosis as measured via ultrasonography, computed tomography (CT), magnetic resonance angiography (MRA), or digital subtraction angiography (DSA), and asymptomatic patients ≥70% stenosis as measured via these same approaches. Patients will be considered symptomatic if they exhibit hemispheric or retinal TIA or non-disabling stroke or retinal infarct within a 6-month period prior to enrollment in this study. All patients must provide written informed consent and must be willing to comply with scheduled visits, treatment plan, laboratory tests, and all other protocols associated with the present study.

Patient will be excluded from this study if they meet any of the following criteria: patients that are undergoing CAS to simultaneously treat acute thrombolysis or mechanical thrombectomy in patients with acute stroke; patients that suffer from non-atherosclerotic carotid disease; patients have undergone any percutaneous interventions or surgeries within the past 30 days; patients that require oral anticoagulant therapy; patients have a high bleeding risk or other antiplatelet therapy contraindications (such as platelet counts < 70 × 10^9^/L); patients that suffer from renal failure and have a serum creatinine level >3 mg/dl; patients that have liver disease or abnormal liver function, with glucosidase or glucosidase levels at least 1.5 times greater than the upper normal limit; patients that exhibit myopathy or creatine kinase (CK) levels at least 2 times greater than the upper normal limit; patients that are females who are pregnant, lactating, or planning to become pregnant during the study period, or who are of child-bearing age and not using acceptable forms of birth control; patients that have pacemakers or who cannot undergo MRI examination due to claustrophobia.

### Randomization

All patients will be randomized into either a routine treatment group or a high-dose atorvastatin group. These patients will be subjected to a stratified blocked randomization method that will assign individuals to treatment groups based upon age(> 70 years or not) and presentation (symptomatic or asymptomatic). Random number generation by a dedicated software program will then be used to assign patients to groups with 1:1 matching grouping and no cross-grouping are permitted.

### Intervention

Individuals in the high-dose group will be administered 80 mg/day atorvastatin beginning 3 days prior to CAS and continuing until 3 days following CAS, whereas routine treatment group patients will receive 20 mg/day atorvastatin during this same period. For the remainder of hospitalization and after discharge, all patients will be administered 20 mg/day atorvastatin. Cerebral protection devices will be required for all patients, but the form of the protective device, the type of stent used, and the interventional strategy will be left to clinical discretion.

Before the CAS procedure, six-vessel DSA is routinely performed. Symptomatic patients with carotid stenosis <50% and asymptomatic patients with carotid stenosis <70% confirmed by cerebral angiography will be excluded from this study.

During the CAS procedure, the distal embolic protection device will be used for all patients. Type of distal protective device (Spider FX., Medtronic; Emboshield NAV, Abbott), carotid stent (Precise, Cordis; Protégé, Medtronic; Acculink, Abbott), pre- and post- dilatation balloon (Ryujin plus, Terumo; Sterling, Boston Scientific, Viatrac, Abbott) will be left to operators' discretion.

Within 5 days prior to CAS, all patients will undergo an MRI examination (including DW-MRI), a neurological assessment, and blood sampling in order to test for parameters such as hematological findings, lipid levels, and hepatic/renal function. In addition, all patients will receive routine standard of care treatment including aspirin (100 mg/day prior to CAS for ≥ 3 days, or a 300 mg loading dose at 6 h prior to CAS) and clopidogrel (75 mg/ day prior to CAS for ≥ 3 days, or a 300 mg loading dose at 6 h prior to CAS). All patients will receive platelet aggregation tests using optical aggregometry before the procedure, and those who show clopidogrel resistance will switching to ticagrelor 90 mg, twice a day. Patients will then undergo routine conventional dual antiplatelet aggregation treatment for 3 months, followed by long-term aspirin administration (100 mg/day). Within 5 days following the CAS operation, all study subjects will again be subjected to DW-MRI and blood tests of the same parameters measured preoperatively.

### MRI Protocol

MRI was performed using a 1.5T imaging unit (Optima MR360, G.E. Medical Systems). Sequences were applied at the same level, with the same slice thickness and same cut number. Slice thickness comprised the cut thickness (5 mm) plus gap (10 per cent). The standard number of slices was 20.

New ischemic lesions in the brain were defined as hyperintense lesions on postintervention diffusion-weighted imaging that were not present on pretreatment MRI. The pre- and post-operative DW-MRI findings will be compared. New ischemic lesions will be recorded based on the following criteria: whether any are present, their localization, their total number, their maximum diameter (with fully separated lesions on the same level or continuous levels being considered to be a single lesion; Ischemic foci localization will be recorded as “ipsilateral,” “contralateral,” or in “posterior circulation” relative to CAS). Two neuroradiologists with more than 10 years of experience, blinded to patient history and intervention procedure information, will independently analyze all MRI images and will discuss and agree upon these results when their judgements are inconsistent. If no consensus could be reached, a third reviewer had the final decision.

### Data Collection

For each patient, the following demographic information will be recorded: age, gender, diabetes history, hypertension status, hyperlipidemia status, smoking status, peripheral arterial disease status, history of prior surgeries, history of previous percutaneous coronary intervention (PCI), and clinical manifestations (including asymptomatic, stroke, and TIA). Key laboratory test results (including blood creatinine, hemoglobin, and blood low-density lipoprotein [LDL] levels) and carotid plaque properties (soft or hard) determined by carotid ultrasound are also collected. The following angiographic and CAS procedure information will be collected, which including (1) aortic arch tortuosity (2) degree and location of extracranial and intracranial arterial stenosis (3) ulcer plaque and fresh thrombus in the stenotic carotid lesion (4) side of CAS procedure (5) type of embolic protection device (7) type, diameter and length of the carotid stent (8) balloon size, length, inflation pressure, duration as well as numbers of pre- and post- dilatation (9) procedure duration (10) procedure-related adverse events (including intraoperative and postoperative hemodynamic instability (11) intraoperative neurological deterioration (12). Other procedures in the same session, such as vertebral artery stenting and intracranial aneurysm embolization, etc.

### Outcomes

The primary outcome for this study will be the cumulative incidence of new ischemic lesions on post-CAS cerebral DW-MRI, TIA or ischemic stroke within 30 days after CAS. For this analysis, stroke is defined as any neurological deficits that persist for over 24 h by a vascular cause, including cerebral infarction, intracerebral hemorrhage (ICH), and subarachnoid hemorrhage (SAH). TIA is defined as any neurological deficit (cerebral or ocular) that is newly evident and consistent with focal cerebral ischemia but that resolved within a 24 h period (including localized muscular weakness, language dysfunction, transient monocular blindness, or the need for assistance while walking.

In addition, this study will have the following secondary outcomes: (1) the incidence of new ischemic lesions as detected via cerebral DW-MRI within the period of 5-day following CAS; (2) the number of new lesions and the incidence of new lesions >5 mm in size as detected via DW-MRI within this 5-day postoperative period; (3) the incidence of ischemic stroke or TIA within 30 days postoperative period; (4) the incidence of death, any stroke, or myocardial infarction within 30 days postoperative period; (5) rates of bleeding complications, as determined based upon the Thrombolysis In Myocardial Infarction criteria (8), and of entry-site complications (subcutaneous hematoma >10 cm, pseudoaneurysm or arteriovenous fistula).

### Sample Size Estimates

The open-source Open-EPI v3.03 software was used for sample size calculations for the present study. The test level α is 0.05 (two-sided test), and the test efficiency Power (1-β) is 0.08. Based upon findings in the abovementioned meta-analysis and the ARMYDA-9 CAROTID study, we estimated a primary endpoint incidence of 40% in routine-dose control group patients. In contrast, the incidence in the high-dose treatment group, who will undergo therapy longer than did patients in the ARMYDA-9 CAROTID study, was estimated to be lower at 15%. Given a 1:1 control to test group ratio, it was estimated that the control group would require 56 test cases. Given a predicted 15% dropout rates, a required sample size of 65 was calculated for both groups, yielding an overall sample size of 130.

### Statistical Analyses

All categorical variables will be given as percentages, while continuous variables will be means ± standard deviations. Chi-squared tests or Fisher's exact tests will be used to compare categorical variables, whereas continuous variables will be analyzed via *t*-tests or a *U*-tests.

These statistical methods will be used to compare whether there are any differences between the control and test groups at baseline, and whether there are any differences in perioperative ischemic brain injury incidence between these groups. *P* < 0.05 will be the significance threshold. In addition, univariate and multivariate logistic regression analyses will be used as a means of identifying any risk factors associated with the incidence of perioperative ischemic brain injury (primary endpoint and partial secondary endpoint) in CAS patients. Prespecified subgroup analysis will be conducted according to whether age is > 70 years and whether symptomatic or not.

### Patient and Public Involvement

There was no patient or public involvement in the design of this clinical trial protocol.

## Discussion

At present, it remains unclear as to whether patients of Chinese ethnicity undergoing CAS receive the same benefits from high-dose atorvastatin treatment as do patients from Western countries. To date, no published randomized controlled trials (RCTs) addressing this issue have been reported, and as such, the present study is of evident clinical importance.

Atorvastatin is currently the most commonly used drug administered to patients suffering from cardiovascular and cerebrovascular diseases. Its administration is associated with significant reductions in blood lipid levels and with an associated decrease in the risk of myocardial infarction, stroke, and death. Currently, patients with cardiovascular and cerebrovascular diseases, including patients undergoing CAS, are treated with a low (10–20 mg/day) dose of atorvastatin. However, some studies have identified a number of potentially neuroprotective benefits to the administration of a higher 80 mg/day atorvastatin dosing regimen, which is associated with the protection of the vascular endothelium, blood vessel dilation, and reduced risk of thrombosis. In individuals with ischemic cerebrovascular disease, the stroke prevention by aggressive reduction in cholesterol levels (SPARCL) trial, which assessed the ability of intensive atorvastatin treatment to prevent stroke recurrence, confirmed that an 80 mg/day atorvastatin dose was able to effectively prevent the recurrence of stroke and adverse cardiac events in patients that have suffered from TIA or stroke (9). To date, the atorvastatin remains the only statin that has been shown to effectively prevent stroke in RCT studies.

To date, relatively few studies have thoroughly examined statin use in patients undergoing CAS. Recent non-randomized studies have found that the use of statins may be associated with reduced rates of adverse events including TIA, stroke, myocardial infarction, new ischemic foci formation, and death following CAS when comparing patients using statins to those not using statins. Similarly, randomized studies have found statin used to be associated with lower rates of stroke, TIA, new ischemic foci formation, myocardial infarction, and death following CAS (10, 11).

Studies of Western populations have observed a 17% incidence of new ischemic lesions in patients treated with 120 mg atorvastatin prior to CAS, as detected via DW-MRI (7). Additional research suggests that micro-emboli detachment can still occur 1 day post-CAS. As such, the use of atorvastatin for a longer duration both pre- and post-CAS may be associated with lower rates of new ischemic lesion formation. As previous studies have demonstrated that 3 days of continuous oral dosing with atorvastatin is sufficient to achieve steady-state plasma concentrations in patients with significant reductions in LDL levels in treated patients (12, 13), we plan to provide patients in this study with a loading dose regimen wherein they begin taking an oral loading dose of atorvastatin 3 days prior to CAS and continue to do so for 3 days postoperatively. The goal of this approach is to take into account both the safety of patients and the practicality of implementing such a dosing regimen.

## Ethics Statement

The studies involving human participants were reviewed and approved by Beijing Hospital Ethics Committee. The patients/participants provided their written informed consent to participate in this study.

## Author Contributions

HW, JL, and JW contributed to the concept and design of this study and this manuscript, drafted the manuscript, revised the manuscript based on co-author feedback, and approved the final version for submission. DW contributed to the concept and design of this study and this manuscript, reviewed the manuscript, and approved the final version for submission. All authors contributed to the article and approved the submitted version.

## Conflict of Interest

The authors declare that the research was conducted in the absence of any commercial or financial relationships that could be construed as a potential conflict of interest.

## Appendix

**Table:** **Trial registration data set**.

**Data category**	**Information**
Primary registry and trial identifying number	ClinicalTrials.gov NCT03079115
Date of registration in primary registry	March 14, 2017
Secondary identifying numbers	121-2016006
Source (s) of monetary or material support	Beijing Hospital
Primary sponsor	Beijing Hospital
Secondary sponsor (s)	
Contact for public queries	Xin Wang wangxinannie@126.com
Contact for scientific queries	Jun Lu, M.D. frente.lu@hotmail.com
Public title	Efficacy of Two Different Doses of Atorvastatin for Prevention of Periprocedural Ischemic Brain Damage in Chinese Patients Undergoing Carotid Artery Stenting (CAS)
Scientific title	Effects of High dose of atorvastatin for Preventing periprocedural Ischemic brain damage in patients undergoing Carotid Artery Stenting (PICAS) in China: A Randomized Controlled Clinical Trial
Countries of recruitment	CHINA
Health condition (s) or problem(s) studied	Carotid Stenosis, Carotid artery stenting, High-dose statin therapy
Intervention (s)	High-dose Atorvastatin Arm: (80 mg/day from 3 days before to 3 days after CAS, and thereafter 20mg/day until 30 days after CAS) Conventional-dose Atorvastatin Arm: (20 mg/day from 3 days before to 30 days after CAS).
Key inclusion and exclusion criteria	Ages Eligible for Study: 40 Years to 90 Years (Adult, Older Adult) Sexes Eligible for the study: both Inclusion criteria: ≥ 50% stenosis of the internal carotid artery in symptomatic patients; or ≥ 70% stenosis of the internal carotid artery in asymptomatic patients received statin therapy for ≥ 2weeks before inclusion Exclusion criteria: nonatherosclerotic carotid disease (dissection, radiation-induced stenosis) received endovascular procedure within 30 days before inclusion CAS during the procedure of urgent endovascular therapy for acute ischemic stroke
Study type	Interventional (Clinical Trial) Allocation: randomized Intervention model: parallel assignment Masking: single(outcomes assessor) Primary purpose: treatment
Date of the first enrolment	August 21, 2017
Target sample size	130 participants
Recruitment status	Recruiting
Primary outcome (s)	Cumulative incidence of new ischemic lesion on post-CAS cerebral DW-MRI, TIA or ischemic stroke within 30 days after CAS
Key secondary outcomes	incidence of new ischemic lesion on post-CAS DW-MRI; number of new lesions on post-CAS DW-MRI; incidence of new lesion > 5 mm on post-CAS DW-MRI
